# Supplementary material for: The Influence of Air Nanobubbles on Controlling the Synthesis of Calcium Carbonate Crystals
Source: Materials (Basel). 2022 Oct 23;15(21):7437. doi: 10.3390/ma15217437 (PMC9655898; doi:10.3390/ma15217437)
Supplement: Supplementary file 1 [file materials-15-07437-s001.zip › materials-1977433-supplementary.pdf]

materials

MDPI

Supplementary Materials

The Influence of Air Nanobubbles on Controlling the Synthesis of Calcium Carbonate Crystals

Yongxiang Wu [1], inyi Huang [1], Chunlin He [1], Kaituo Wang [1], Nguyen Thi Hong Nhung [1], Siming Lu [2], Gjergj Dodbiba [3], Akira Otsuki [4,5] and Toyohisa Fujita [1,*]

Citation: Wu, Y.; Huang, M.; He, C.; Wang, K.; Nhung, N.T.H.; Lu, S.; Dodbiba, G.; Otsuki, A.; Fujita, T. The Influence of Air Nanobubbles on Controlling the Synthesis of Calcium Carbonate Crystals. *Materials* **2022**, *15*, 7437. https:// doi.org/ 10.3390/ma15217437

Academic Editor: Carlos Javier Duran-Valle

Received: 2 October 2022 Accepted: 20 October 2022 Published: 23 October 2022

Publisher's Note: MDPI stays neu-tral with regard to jurisdictional claims in published maps and institu-tional affiliations.

Copyright: © 2022 by the authors. Li-censee MDPI, Basel, Switzerland. This article is an open access article distributed under the terms and con-ditions of the Creative Commons At-tribution (CC BY) license (https://cre-ativecommons.org/licenses/by/4.0/).

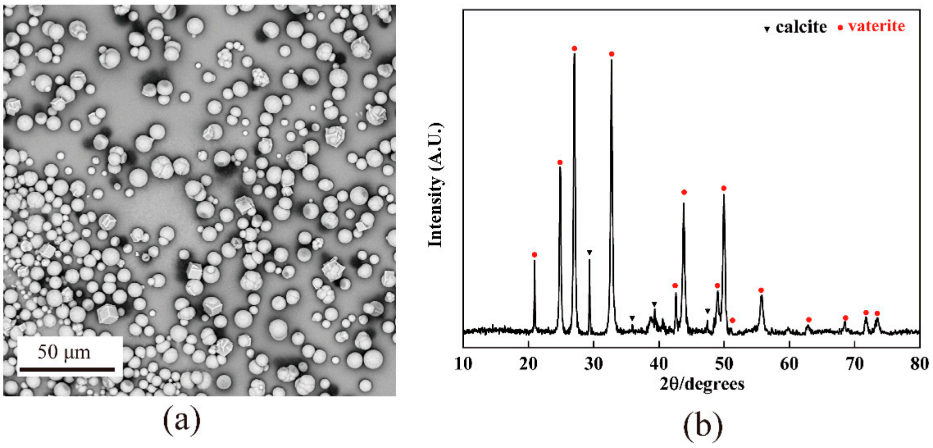

Figure S1. SEM images and XRD parts of vaterite obtained

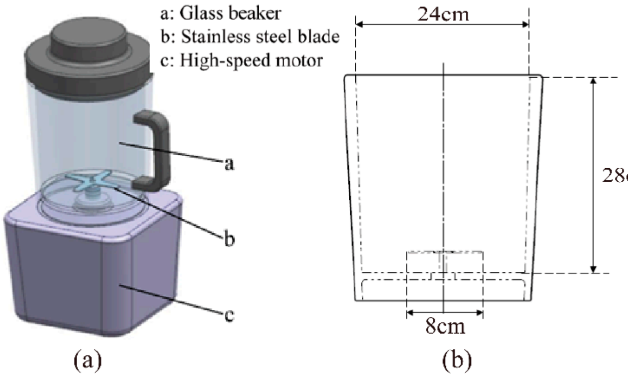

Figure S2. The schematic diagram of the machine for producing bulk nanobubbles(a), and the size of the stain-less-steel blade and beaker (b).

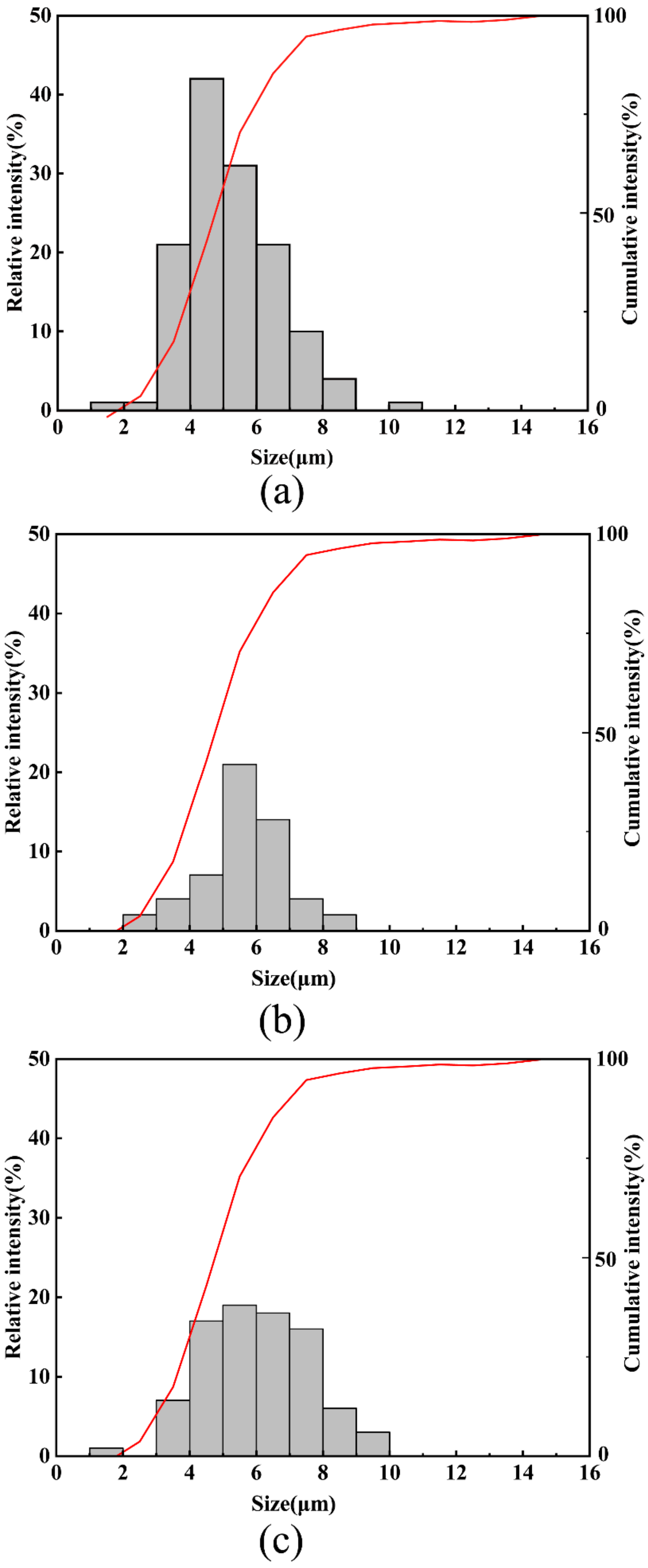

Figure S3. Size distribution of calcite after 4 hours (a), size distribution of calcite after 1 hours, and size distribution of vaterite after 1 hours prepared in 2.96×10^8 particles/mL of nanobubbles water.

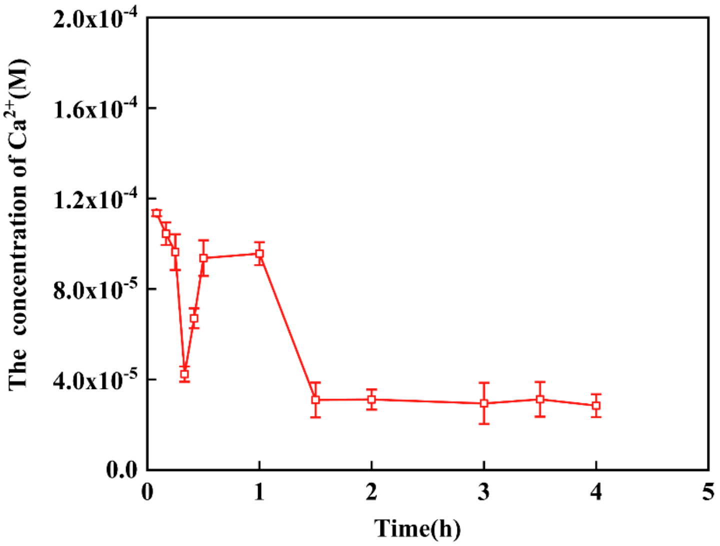

Figure S4. The concentration of Ca^{2+} depending on the time in the solution filtrated at 0.45 μm after the reaction to prepare $CaCO_3$
